# EOS imaging and scoliosis: the clinical applicability and intra-rater repeatability of measures

**DOI:** 10.1007/s00256-025-05020-2

**Published:** 2025-09-01

**Authors:** Matthew Bellamy, Raveen Jayasuriya, Lee Breakwell, Ashley Cole

**Affiliations:** 1 The Medical School, The University of Sheffield, Sheffield, South Yorkshire UK; 2 Department of Paediatric Orthopaedics, Trauma and Spinal Surgery, Sheffield Children’s Hospital NHS Trust, S10 2TH, Clarkson Street, Broomhall, Sheffield, South Yorkshire UK

**Keywords:** EOS imaging, Three-dimensional reconstruction, Repeatability, Intraclass correlation coefficient, Technical error of measurement

## Abstract

**Objectives:**

EOS bi-planar imaging enables three-dimensional (3D) reconstructions of the spine and pelvis with segmental vertebral measurements in three planes from a neutral pelvis. This study evaluates the repeatability of these measurements and the accuracy in detecting true changes.

**Methods:**

Twenty patients from four clinical backgrounds (surgical threshold, bracing threshold, micro-dose, and in-brace) were included. EOS bi-planar “full spine” images were modelled and then subsequently re-modelled at least 4 weeks later by the same researcher. All 3D measurements were recorded and compared.

**Results:**

The average modelling interval was 6.7 weeks. Paired measures indicated high agreement, except for planes of maximal curvature (PMC): thoracic (Spearman’s = 0.67; *p* < 0.05) and lumbar (Spearman’s = 0.40; *p* > 0.05). Intraclass correlation coefficients (ICCs) showed excellent agreement, with thoracic and lumbar Cobb angles averaging 0.99. Sagittal measurements ranged from 0.93 (L1/S1 lordosis) to 0.96 (T1/T12 kyphosis). Pelvic parameters ranged from 0.88 (obliquity) to 0.99 (tilt). The transverse profile ranged from 0.82 (apical thoracic rotation) to 0.98 (average lumbar rotation). Repeatability (2.77 × technical error of measurement [TEM]) was ± 4.4° for Cobb angles, ± 7.7° for sagittal profile, ± 5.0° for pelvic parameters, ± 4.8° for transverse profile, and ± 100.4° for automated thoracic and lumbar PMC. With strong outliers excluded, thoracic PMC was ± 16.2° and lumbar PMC was ± 15.5°.

**Conclusion:**

3D EOS measurements demonstrate excellent intra-rater ICC repeatability despite notable true measurement error that should define future success criteria. Semi-automated modelling provides quick 3D spinal alignment measurements from a neutral pelvis, with this study being the first to report TEM for 3D EOS reconstructions. PMC disagreement indicates the need for further investigation.

## Introduction

In 1992, George Charpak won a Nobel Prize with his new gas particle detector, allowing the conversion of photons into electrons when exposed to a pressurised gas such as xenon [1]. The number of photons generated increased through this detector, whilst the amount of radiation exposure is minimised [1]. This system has been progressed and is now known as the EOS imaging system.

Benefits from this EOS system stem from the radiation dose reduction and the ability to take simultaneous bi-planar images whilst the patient is standing in a weight-bearing position [1]. EOS imaging reduces the radiation dose by up to 10 times compared to standard X-rays [2]. To further reduce the radiation delivered by EOS imaging, a novel micro-dose setting was developed. This delivers 5.5 times less radiation than EOS full dose and 45 times less radiation than a standard X-ray [3].

Due to the pre-calibrated simultaneous bi-planar images, a new method for three-dimensional modelling was developed. This method relies on positioning an overlaid vertebral anatomical map onto the already acquired EOS X-rays to mark out bone contours, alignment, and rotation for each point of interest [4]. A 2013 paper showed that EOS 3D modelling provides accurate 3D models comparable to those taken from a computed tomography (CT) scan [5]. Bi-planar, clear visualisation of spinal deformity with a reduction in radiation dose is especially important in a paediatric population.

Somoskeoy et al. studied 201 patients with scoliosis and 10 patients without scoliosis. In their study, three different examiners determined coronal or sagittal curve parameters from either the manual 2D measurements or the automated 3D measurements given after modelling. They found that 3D measurements gave non-significant differences compared to 2D measurements. The intra-observer reliability was high for both methods, with inter-rater reproducibility being higher in the 3D models [6].

This study aimed to assess the repeatability of all global spinal measurements taken from EOS 3D models, with emphasis on the measurements from the transverse plane.

## Methods

### Training and planning

To learn the radiographic and modelling functions of EOS imaging, an intensive online course was undertaken across the space of four weeks. This online course, which spanned around 4 to 6 hours of learning the dedicated written material, provided training in both core and advanced workflows for EOS spinal procedures. It included multiple online video modules and practical online sessions, covering the generation of 3D models from T1 to L5 and the full spinal modelling process with pelvic parameters. Successful completion was validated through a post-course examination, leading to certification of the acquired skills. This was then supplemented by several local practice sessions for the remainder of the month in EOS generation modelling. The development and implementation of this study were approved by the NHS Health Research Authority and by the local clinical research department (HRA:4632, IRAS:321532 ). Approval from the local Institutional Review Board was obtained, and in keeping with the policies for a retrospective review of routine clinical scans, informed consent was not required from participants. No funding was obtained for this study.

### Settings and participants

Inclusion criteria were simultaneous posteroanterior (PA) and lateral EOS images, as well as images spanning from the internal auditory meatus to the femoral heads. There was no restriction on age, curvature size, or aetiology of disease, with idiopathic and non-idiopathic curves modelled. Exclusion criteria were PA-only radiographs, images where the last cervical vertebra or the femoral heads were not visible, patients who had undergone scoliosis correction or any other surgery, patients with congenital or developmental abnormalities of the spine, patients with motion artefacts on imaging, or very abnormally shaped vertebral bodies.

### Patients and EOS image generation

**Fig. 1:**
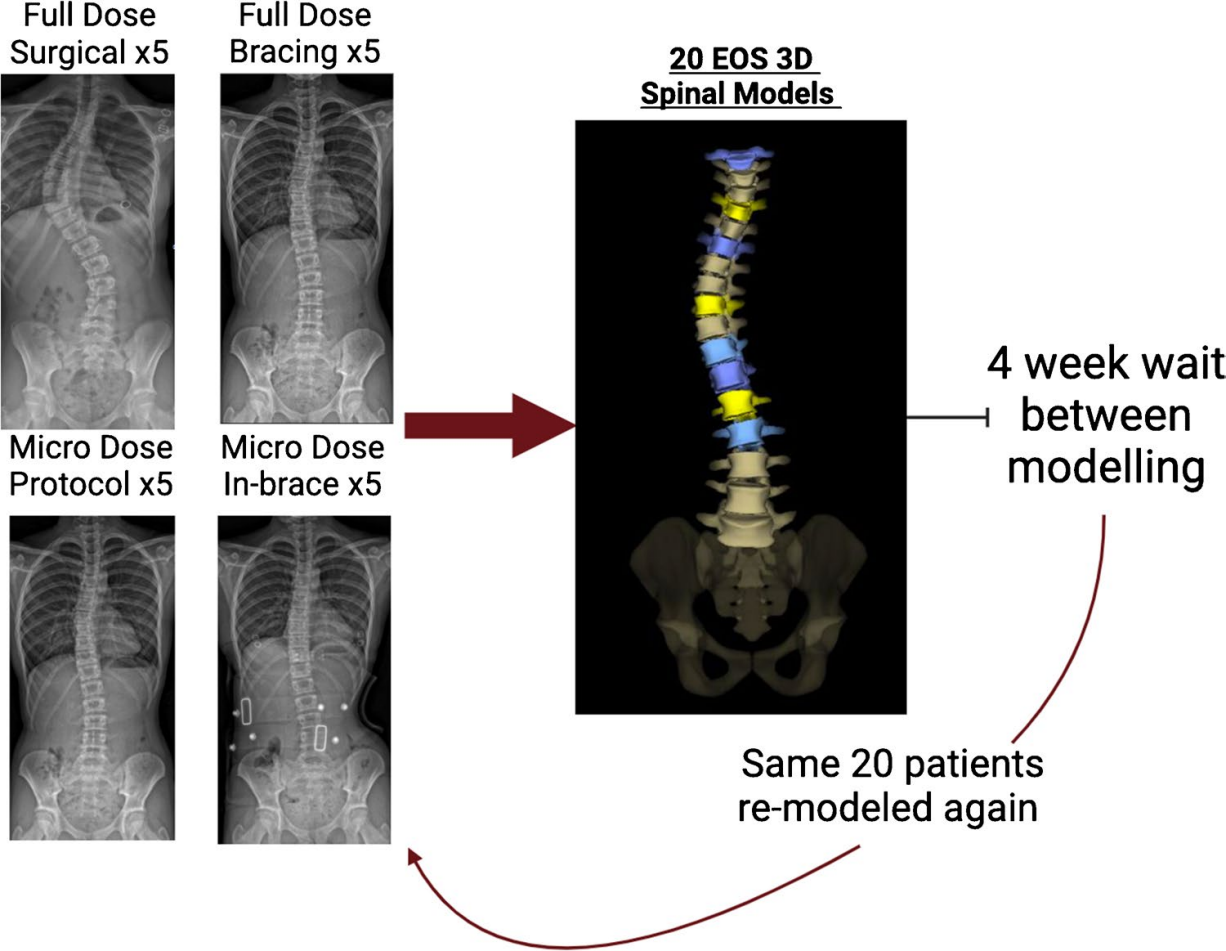
Repeatability timeline for 20 patients’ 3D EOS models recreated at two different time points, at least 4 weeks apart, by the same observer

Patients were selected from each of four different groups to create a cohort representative of our normal clinical population (Fig. 1). All selected patients had their 3D models generated from bi-planar X-rays by a single, trained observer. The study utilised retrospective data from patients who had previously undergone bi-planar EOS imaging as part of their routine clinical care. Before generation of the second model, a minimum of 4 weeks was observed to ensure local recall of anatomy was avoided. After this time, the same patients had their 3D models generated for a second time by the same observer. The EOS imaging system and sterEOS 3D modelling software display patient names during the modelling process, preventing anonymisation at the time of 3D reconstruction. This meant that patient data could only be anonymised after the modelling phase was complete.

### Materials and data collection

Although this study assesses the repeatability of the measurements, for 3D EOS models, radiographic angles are generated automatically once the model has been reconstructed, so it is effectively a repeatability of generating the 3D EOS models. The reconstructed 3D models were used to extract all measurements displayed in Table 1.

**Table 1:** Specific spinal measures across all 3 planes extracted from the 3D EOS imaging models

Coronal	Sagittal	Transverse	Global	Pelvic
Thoracic Cobb angle	T1/T12 kyphosis	T1 to L5 individual rotations	Thoracic plane of maximal curvature	Incidence
Lumbar Cobb angle	T4/T12 kyphosis	Rotation of the apical vertebra		Obliquity
Apical vertebra level	L1/S1 lordosis	T1 to T6 average rotation	Lumbar plane of maximal curvature	Sacral slope
	L1/L5 lordosis	T7 to T12 average rotation		Tilt
		L1 to L5 average rotation		Pelvic axial rotation

Measurement definitions: Thoracic Cobb angle, magnitude of the thoracic spinal curve; lumbar Cobb angle, magnitude of the lumbar spinal curve; apical vertebra level, most laterally displaced vertebra in a curve; T1/T12 kyphosis, outward curve of the entire thoracic spine; T4/T12 kyphosis, outward curve of the lower thoracic spine; L1/S1 lordosis, inward curve of the entire lumbar spine; L1/L5 lordosis, inward curve of the main lumbar spine; T1 to L5 individual rotations, rotation of each vertebra from T1 to L5; rotation of the apical vertebra, specific rotation of the most laterally displaced vertebra; thoracic plane of maximal curvature, vertical plane connecting the mid-points of the end vertebrae of the thoracic curve and the mid-point of the thoracic apical vertebra; lumbar plane of maximal curvature, vertical plane connecting the mid-points of the end vertebrae of the lumbar curve and the mid-point of the lumbar apical vertebra; incidence, angle between a line perpendicular to the sacral endplate at its midpoint and the midpoint of the line connecting the centre of each acetabulum (pelvic incidence = pelvic tilt + sacral slope); obliquity, frontal plane angle of the line connecting the highest point of each acetabulum to the horizontal; sacral slope, angle of the sacrum relative to a horizontal line; tilt, angle between the line connecting the mid-point of the sacral endplate to the midpoint of the acetabular axis and the vertical line; pelvic axial rotation, in the axial plane, the angle between the acetabular axis and the frontal plane

### Statistical analysis

The statistical analysis was performed using the statistical software SPSS v28.0 (IBM, 2023) by the lead researcher (MB). Statistical significance was accepted at a *p*-value less than 0.05.

Due to the sample size, all measurements were treated as non-parametric variables. Correlation in measurements between the first and second models was analysed using Spearman’s rank. ICCs were calculated and summarised separately for each continuous variable measured by 3D models. A two-way mixed-effects model for single measures was applied for estimation of ICC and 95% confidence intervals, due to only one rater reproducing the models, who was not chosen from a random sample. Cicchetti gives the following often-quoted guidelines for interpretation of ICC inter-rater agreement measures [7]:

- Lower than 0.40—poor.

- In-between 0.40 and 0.59—fair.

- In-between 0.60 and 0.74—good.

- In-between 0.75 and 1.00—excellent.

The technical error of measurement (TEM) was also calculated to determine repeatability measurements [8].

Technical error of measurement (TEM) = $\surd (\Sigma d2 / 2n)$

where *d* = difference between two measures and *n* = number of subjects.

The difference between a subject’s measurement and its true value is 1.96 multiplied by the TEM. However, the difference between two measurements on the same subject is termed ‘repeatability’ and is calculated by multiplying TEM by 2.77 (1.96 × square root of 2). The subject’s measure will be within the TEM multiplied by 1.96 of the actual value 95% of the time. The repeatability, whilst a larger number, is probably clinically the most relevant when considering true measurement error.

## Results

In our study, 20 patients’ radiographs were retrospectively used. Mean age was 14.9 years and mean Cobb angle was 26° (range: 11–71). The female-to-male ratio was 10:1. Average time between the two models was 47 days (6 weeks and 5 days). The minimum time between the two models was 34 days (4.9 weeks). Overall, 16 patients had adolescent idiopathic scoliosis, three had early onset, and one had a syndromic scoliosis. There were no paraspinal abnormalities in the patient with syndromic scoliosis. There were no abnormalities of vertebral body shape necessitating the exclusion of scans.

### Spearman’s correlation

Correlation between the two time points for the Cobb angles averaged 0.98 (*p* < 0.001), indicating a high level of agreement between measures (Table 2). Correlation in the sagittal profile (T1–T12 kyphosis, T4–T12 kyphosis, L1–L5 lordosis, and L1–S1 lordosis) ranged from 0.91 to 0.96 (*p* < 0.01). Correlation in the pelvic measures ranged from 0.80 (pelvic obliquity; *p* < 0.01) to 0.98 (pelvic axial rotation; *p* < 0.01).

**Table 2:** Spearman’s correlation between measurement 1 and measurement 2 for non-parametric EOS data

	Parameter	Spearman’s correlation	Two-sided *P*
Coronal	Thoracic Cobb angle	0.97	< 0.001*
	Lumbar Cobb angle	0.98	< 0.001*
Sagittal	T1/T12 kyphosis	0.94	< 0.001*
	T4/T12 kyphosis	0.91	< 0.001*
	L1/L5 lordosis	0.96	< 0.001*
	L1/S1 lordosis	0.93	< 0.001*
Axial	Thoracic AVR	0.79	< 0.001*
	Lumbar AVR	0.96	< 0.001*
	Average thoracic rotation	0.92	< 0.001*
	Average lumbar rotation	0.98	< 0.001*
	T1–T6 thoracic rotation	0.87	< 0.001*
	T7–T12 thoracic rotation	0.95	< 0.001*
Pelvic	Pelvic incidence	0.93	< 0.001*
	Pelvic obliquity	0.88	< 0.001*
	Sacral slope	0.80	< 0.001*
	Pelvic tilt	0.98	< 0.001*
	Pelvic rotation	0.98	< 0.001*
PMC	Thoracic PMC	0.67	0.02*
	Lumbar PMC	0.40	0.09

In the axial plane, correlation ranged from 0.79 (thoracic apical vertebral rotation (AVR); *p* < 0.01) to 0.98 (average lumbar rotation; *p* < 0.01). The thoracic plane of maximal curvature (PMC) showed a significant correlation of 0.67 (*p* < 0.05). However, the lumbar PMC showed no significant correlation between the two time points, at 0.40 (*p* > 0.05).

### Technical error of measurement and repeatability

The technical error of measurement (TEM) and subsequent repeatability are displayed in Table 3. If the same measurement is taken twice from the same radiographs, 95% of the time, the measurement will be within 2.77 × TEM. The repeatability for thoracic and lumbar Cobb angles was on average ± 4.4°. The measures for the sagittal profile (T1–T12 kyphosis, T4–T12 kyphosis, L1–L5 lordosis and L1–S1 lordosis) averaged ± 7.7°.

**Table 3:** Technical error of measurement and repeatability for each automated variable that EOS imaging produces as part of the ‘full spine’ protocol

Plane	Measure	TEM (degrees)	Repeatability (degrees)
Coronal	Thoracic Cobb angle	+/− 1.5	4.1
	Lumbar Cobb angle	+/− 1.7	4.7
Sagittal	T1/T12 kyphosis	+/− 2.5	7.1
	T4/T12 kyphosis	+/− 2.6	7.1
	L1/L5 lordosis	+/− 2.5	7.0
	L1/S1 lordosis	+/− 3.4	9.5
Apical	Thoracic apical rotation	+/− 3.1	8.7
	Lumbar apical rotation	+/− 2.4	6.6
Average rotations	T1–T12 rotation	+/− 0.9	2.6
	L1–L5 rotation	+/− 1.4	3.8
	T1–T6 average rotation	+/− 0.9	2.6
	T7–T12 average rotation	+/− 2.0	5.4

The transverse profile (apical vertebral rotation, average thoracic rotation, T1–T6 average rotation, T7–T12 average rotation, and average lumbar rotation) had a repeatability of ± 4.8°, whilst the automated thoracic and lumbar PMC gave a repeatability of ± 100.4°. However, this analysis was significantly influenced by one outlier in the thoracic curves and three lumbar curves. With these strong outliers excluded the repeatability for the thoracic PMC was ± 16.2 and ± 15.5° for the lumbar PMC. The PMC was automatically derived from the 3D reconstruction data post-modelling, without direct measurement by the researcher undertaking the modelling. Outliers in PMC measurements were linked to cases where values had often closely similar magnitudes but opposing directions (e.g. positive to negative). Since the TEM calculation involves squaring these differences, the directional discrepancies disproportionately raised the TEM values. For example, even with other consistent lumbar parameters, the PMC was recorded as positive in one case and negative in another. Consequently, these outliers were excluded to provide a more accurate representation of the PMC within the main study cohort.

The pelvic parameters (incidence, obliquity, sacral slope, axial rotation, and tilt) averaged a repeatability of ± 5.0°. The largest outlier was the sacral slope and hence the linked pelvic incidence (pelvic incidence = pelvic tilt + sacral slope) (Table 4).

**Table 4:** Technical error of measurement and repeatability for each automated pelvic parameter that EOS imaging produces as part of the ‘full spine’ protocol

	Measure	TEM (degrees)	Repeatability (degrees)
Pelvic	Pelvic incidence	+/− 3.4	9.5
	Pelvic obliquity	+/− 1.1	3.0
	Sacral slope	+/− 3.3	9.0
	Pelvic axial rotation	+/− 0.5	1.4
	Pelvic tilt	+/− 0.7	2.0

### Intraclass correlation coefficients

**Fig. 2:**
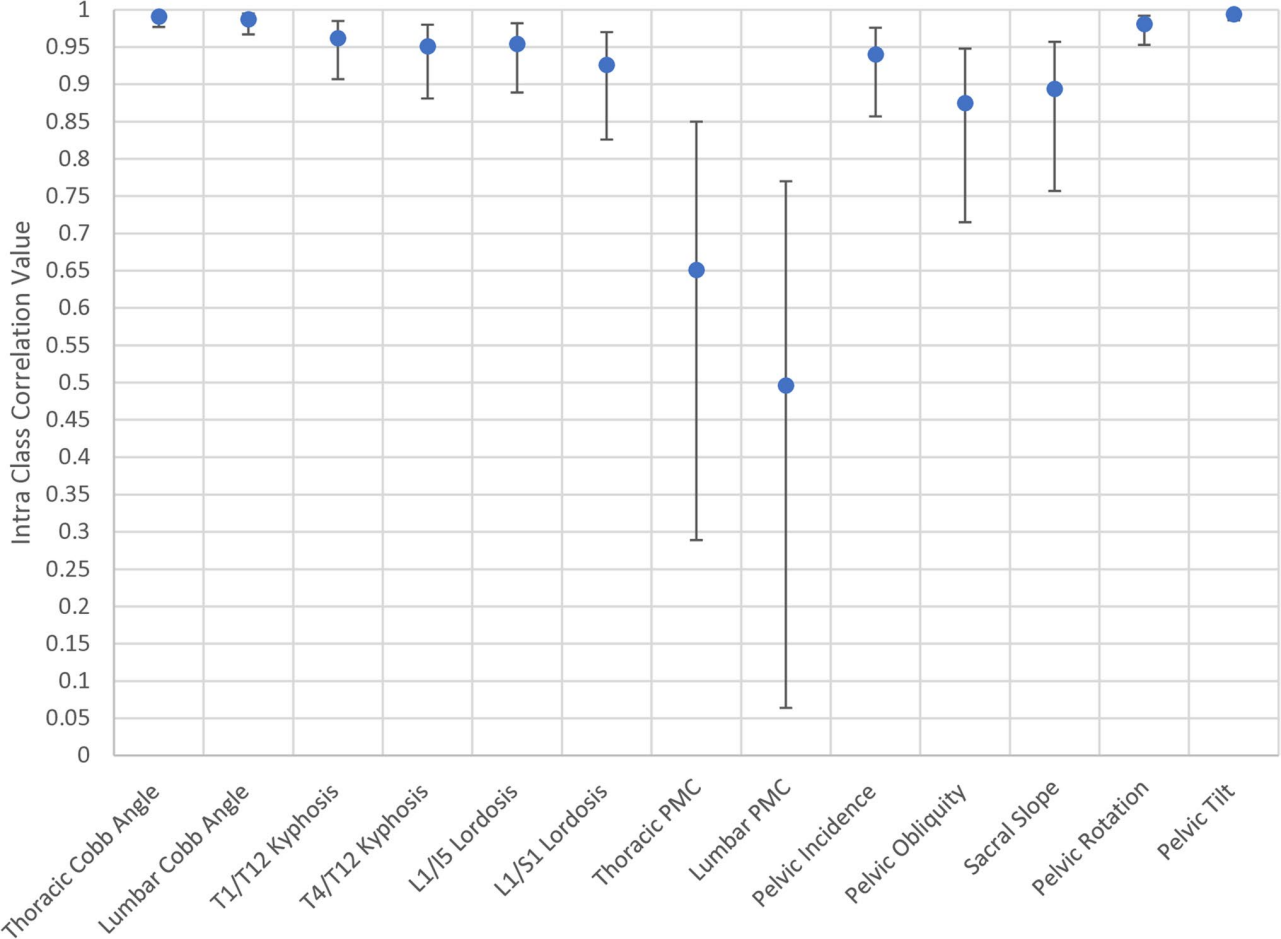
ICC values for global spinal measures. The centre dot represents the ICC value with the associated upper and lower 95% confidence intervals

**Fig. 3:**
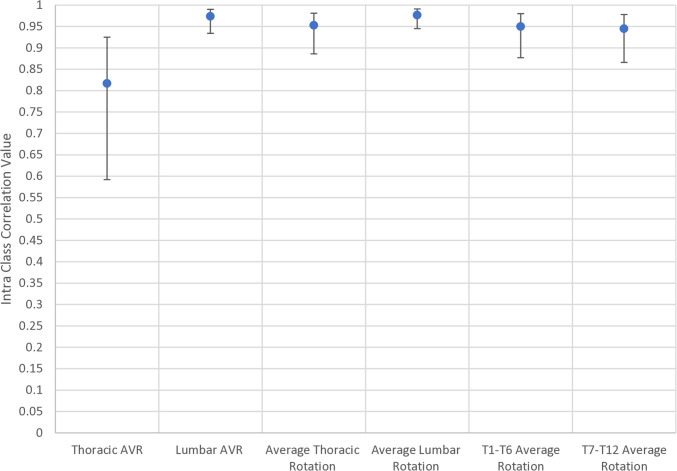
ICC values for global axial plane measures. The centre dot represents the ICC value with the associated upper and lower 95% confidence intervals. AVR, apical vertebral rotation

**Fig. 4:**
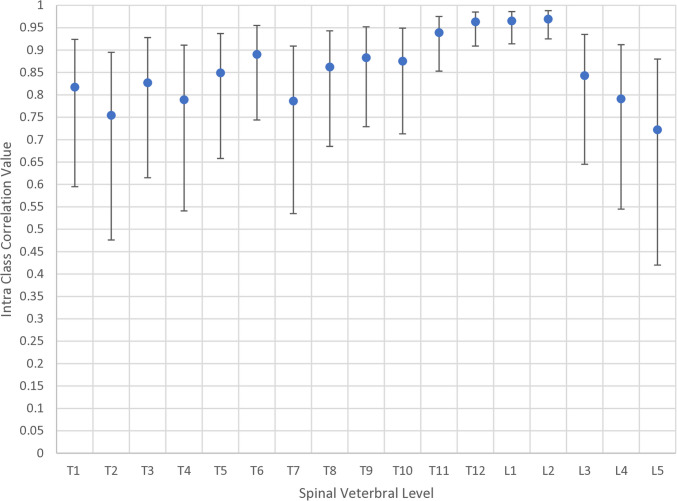
ICC values for individual vertebral axial measures. The centre dot represents the ICC value with the associated upper and lower 95% confidence intervals

A full breakdown of the intraclass correlations (ICC) with 95% confidence intervals for each variable is given in Table 5. This is analysed as a whole group (*n* = 20) (Figs. 2, 3, and 4).

**Table 5:** Intraclass correlation values with the respective 95% confidence intervals

	Whole cohort	95% CI lower	95% CI upper
Thoracic Cobb angle	0.99	0.98	0.99
Thoracic AVR	0.82	0.59	0.93
Lumbar Cobb angle	0.99	0.97	0.99
Lumbar AVR	0.97	0.93	0.99
T1/T12 kyphosis	0.96	0.91	0.99
T4/T12 kyphosis	0.95	0.88	0.98
L1/L5 lordosis	0.95	0.89	0.98
L1/S1 lordosis	0.93	0.83	0.97
T1–T12 average rotation	0.95	0.89	0.98
L1–L5 average rotation	0.98	0.95	0.99
T1–T6 average rotation	0.95	0.88	0.98
T7–T12 average rotation	0.95	0.87	0.98
Thoracic PMC	0.65	0.29	0.85
Lumbar PMC	0.50	0.06	0.77
Pelvic incidence	0.94	0.86	0.98
Pelvic obliquity	0.88	0.72	0.95
Sacral slope	0.89	0.76	0.96
Pelvic axial rotation	0.98	0.95	0.99
Pelvic tilt	0.99	0.99	0.99

ICC values range from 0 to 1, with a value close to 1 indicating good repeatability of measures. *AVR*, apical vertebral rotation; *PMC*, plane of maximal curvature

The ICC for thoracic and lumbar Cobb angles was 0.99 respectively. ICC for the sagittal profile (T1–T12 kyphosis, T4–T12 kyphosis, L1–L5 lordosis, and L1–S1 lordosis) ranged from 0.93 (L1/S1 lordosis) to 0.96 (T1–T12 kyphosis). ICC for pelvic parameters (pelvic incidence, pelvic obliquity, sacral slope, pelvic axial rotation, and pelvic tilt) ranged from 0.88 (obliquity) to 0.99 (tilt). ICCs in the transverse profile (apical vertebral rotation, average thoracic rotation, T1–T5 average rotation, T6–T2 average rotation, and average lumbar rotation) ranged from 0.82 (thoracic apical rotation) to 0.98 (average lumbar rotation).

## Discussion

This repeatability analysis is important to assess and validate the present and future work in this field using 3D models from EOS imaging. Previously, 3D models have been provided by CT imaging. However, the large radiation exposure and ‘supine-only’ imaging make this an infrequently used tool in paediatric spinal deformity. Measurements from EOS imaging have been shown to be comparable to that of CT scans with the benefit of low radiation exposure [5]. Parameters such as vertebral rotation and pelvic alignment are becoming increasingly important in scoliosis evaluation and follow-up. In young patients who will be treated for several years, minimising the radiation dose while obtaining accurate clinical measures remains a high priority.

This repeatability analysis demonstrates that the first and second 3D models were significantly correlated apart from the lumbar plane of maximal curvature. The largest differences were seen in the lumbar plane of maximal curvature; however, this is an average measure across the global alignment and so is more prone to larger intrinsic errors. We also note that although first proposed to be a first step in the direction of gaining a new 3D classification of scoliosis, there has been no validation or reliability work looking at the plane of maximal curvature [9]. With large magnitudes of variation seen in this initial analysis, larger studies looking at the accuracy, repeatability, and agreement between PMC measures are vital before integration into routine care.

No repeatability using the TEM has been done with 3D spinal reconstructions generated from EOS imaging. Another study in 2011 looked at pre- and post-operative repeatability using the mean average of variance across three raters, which is similar, but not the same as calculating a TEM [10]. Comparing this repeatability study to theirs, we see that both the thoracic and lumbar Cobb angles had a confidence of around 4 to 5° when calculated using variance. The repeatability in our study when calculated with 2.77 × TEM for Cobb angle measurements is similar at between 4 and 5° (4.1–4.7). This study has a larger repeatability variation in sagittal measures (7.0–9.5°) than in the earlier study (4.4–5.9°). This study also had a larger range for both thoracic and lumbar AVR measurements (8.7 and 6.6°), indicating a large uncertainty on the significance of the rotations. However, in the previous study, the pre-operative AVR had a reliability of 5.3° for the intra-rater reliability and 6.1° across all three raters, showing that significant variation in apical rotation measurement is not limited to this study and may lead to inaccuracy in rotation reporting after EOS 3D modelling. It is also worth noting that in the previous study, all three operators were either experienced with EOS 3D models or were senior spinal surgeons with experience in spinal anatomy and radiography.

We also see a large measurement error in the sacral slope and subsequently the pelvic obliquity, relying on sacral end plate clarity. Other studies have also found the sacral slope to be the most unreliable measurement across pelvic parameters [11]. In a recent similar study, for individual raters, the uncertainty in the sacral slope was near 13.9° with the largest variation in bias and variance. However, the ICC remained high (ICC: 0.99). Registration of the sacral slope relies on selecting two small points at each end of an often-blurred sacral endplate. Comparing this to matching the shape of a sphere to the shape of a large acetabulum, the small point measure over a large area is prone to much larger differences [12].

It is worth noting that although the isolated apical vertebra rotation has a larger measurement error, when the rotation is grouped for T1–T6, T7–T12, T1–T12, and L1–L5 vertebra, the measurement error significantly decreases and hence these may be more accurate and clinically useful. A repeatability of over 5° (TEM: 1.8) limits the clinical applicability of these measures. However, when assessing patients for surgical correction or treatment with an orthosis, quantification of the average structural or non-structural curve rotation may prove clinically useful to produce a balanced and even spinal alignment, which may be hard to quantify on an isolated PA X-ray with a rotated pelvis.

All ICC measures, except thoracic and lumbar PMC, were above 0.8, indicating excellent repeatability between measures. The ICC in the upper thoracic and lower lumbar spine was lower than in the lower thoracic and upper lumbar spine, reflecting the difficulties in locating the anatomical landmarks in these regions of the spine. Assessments of the sagittal balance using 3D EOS models have shown good repeatability in other studies, with these results being similar. Comparing the intra-rater reliability to a similarly designed study in 2018, we see that the reported ICC for both thoracic and lumbar Cobb angles was improved in this study [13]. We also see that this study population has a higher ICC value for axial plane parameters. AVR parameters in the previous study ranged from 0.55 to 0.75, with this study improving to 0.82 and 0.97 for both measurements.

Presented axial rotation correlations from 3D EOS modelling are also improved compared with those found by Rehm in his 2017 paper using more than one rater (Table 6) [14]. However, their study had a larger number of patients (*n* = 74), but a smaller Cobb angle on average (18°). Their T1–T6 ICC values ranged from 0.51 to 0.71, with the T7–T12 rotations ranging from 0.59 to 0.81. In this study, these ranged from 0.75 to 0.89 and 0.79 to 0.96 respectively. Thoracic AVR and T1–T11 rotation have a weaker correlation than lumbar AVR and T11–L4 rotation due to the ribs making thoracic pedicle identification more difficult.

**Table 6:** Comparison of this study with 4 different repeatability studies looking at the ICC from EOS 3D models

	Bellamy et al. (2025)	Carreau et al. (2014) [15]	Bagheri et al. (2018) [13]	Ilharreborde et al. (2011) [16]	Rehm et al. (2016) [14]	
Thoracic Cobb angle	0.99	0.98*	0.82	0.99*	N/A	
Thoracic AVR	0.82	0.98*	0.66	0.97*	N/A	
Lumbar Cobb angle	0.99	0.98*	0.91	0.99*	N/A	
Lumbar AVR	0.97	0.98*	0.65	0.97*	N/A	
T1/T12 kyphosis	0.96	0.97	0.87	0.99	0.92	
L1/L5 lordosis	0.95	0.95	0.82	0.98	0.90	
Pelvic incidence	0.94	0.98	N/A	0.99	0.97	
Sacral slope	0.89	0.96	N/A	0.98	0.96	
Pelvic tilt	0.99	0.99	N/A	1.00	0.98	

*AVR*, apical vertebral rotation. * indicates specification of whether thoracic and lumbar measurements were not made

ICC measurements from EOS imaging appear to have good consistency in measurements, especially in the lumbar spine where the anatomy is clearer. These results appear to align with previous literature, offering promising results in the ability to quickly learn the technique of EOS imaging and sterEOS to create 3D spinal models. The high ICC and reasonable measurement repeatability suggest that these models can be accurately reproduced by undertaking an online course with learning over a few months. This expands the ability to model more patients’ global 3D alignment without prolonged training.

Overall, we found that the “full spine” 3D models from EOS imaging had good repeatability and consistency across 20 patients, considering the heterogeneity of the patient population. We found that ICC values for the whole cohort were very similar to the initial work in this field, but previous literature has only reported AVR as one value, making it difficult to compare the thoracic and lumbar rotations exactly. More accuracy and repeatability analysis is needed to determine the role of the PMC in clinical use.

There are some limitations that need to be mentioned. We commenced this research trial after 6 months of using EOS images to determine protocols and applicability, therefore these results cannot be generalised to a rater who has not used the software previously. Due to the nature of the research project, the 3D models were only undertaken by one researcher and hence inter-rater reliability could not be assessed. The heterogeneous aetiology of patients in this study was used to mimic clinical practice. However, this could lead to larger errors in larger or more complex curves. Larger, more homogeneous studies are needed to improve the precision and generalisability of findings, especially concerning the upper thoracic and lower lumbar spine. These studies should focus on addressing the challenges of accurately identifying the plane of maximal curvature and assessing the significance of apical vertebral rotation in these more complex regions.

## Conclusion

Measurements from EOS imaging have shown acceptable intra-rater reliability for most measurements with a low risk of bias between measurements. Measurements in all three planes have shown good to excellent agreement with ICC, apart from both the thoracic and lumbar PMC, where we found high levels of disagreement warranting further investigation before being integrated into routine clinical care. In this study, we found small measurement errors in most pelvic parameters and Cobb angles. However, there was substantial variation in the TEM for sagittal and axial planes, translating into the repeatability, which should define the definitions of success when reporting changes measured on reformatted 3D models in future studies and clinical practice. Measuring average rotations in regions of the spine has a lower TEM and repeatability, potentially making them better in situations where change in rotation is needed over time or after treatment.

## Data Availability

The data and analysis that support the results of this study are available from the corresponding author, M Bellamy, upon reasonable request.

## Abbreviations

ICC: Intraclass correlation coefficient
TEM: Technical error of measurement
CT: Computed tomography
PMC: Plane of maximal curvature
2D: Two-dimensional
3D: Three-dimensional
PA: Posteroanterior
AVR: Apical vertebral rotation
